# Statistical analysis of ventricular shape of ARVC patients and correlation with clinical diagnostic indices

**DOI:** 10.1186/1532-429X-17-S1-P283

**Published:** 2015-02-03

**Authors:** Kristin McLeod, Jørg Saberniak, Kristina Haugaa

**Affiliations:** Cardiac Modelling, Simula Research Laboratory, Lysaker, Norway; Asclepios Research Team, INRIA, Sophia Antipolis, France; Rikshospitalet, Oslo University Hospital, Oslo, Norway

## Background

Arrhythmogenic right ventricular cardiomyopathy (ARVC) is an inherited cardiomyopathy characterized by fatty and fibrotic replacement of cardiac tissue, which ultimately affects the structure, function and electrical propagation of the ventricles. Diagnosis of ARVC is challenging and is currently guided by the 2010 Task force criteria (2010TFC), which includes criteria identified from imaging, ECG and family history.

We aimed to compute a mean 3D model of the ventricles of ARVC patients and analyse the shape modes around this mean to correlate with the 2010TFC indices.

## Methods

We studied 28 ARVC patients fulfilling the 2010TFC ARVC diagnosis criteria at varying stages of the disease retrospectively from cine MRI images acquired from a Siemens SonataVision 1.5T scanner. The mean of these patients was computed using an iterative minimization approach with currents to represent ventricle surfaces and the LDDMM algorithm to compute the pair-wise shape deformations between each patient and the mean. Principal component analysis (PCA) was applied to the mean-to-patient deformations (which encode the shape variation in the population), in order to establish the dominant shape patterns present in this population. The correlations between the PCA shape modes and 11 clinical indices (including the 2010TFC indices) were computed.

## Results

The computed mean ventricular surfaces are shown in Fig. (a) with the patient surfaces overlaid to visualize the shape variability present in the population. In order to capture 90% of the shape variability in the population, 12 modes were required. Shape mode 2 was found to be significantly correlated (p<0.01) to clinical index 8 (the number of ECG major criteria) with p=0.0039. Shape mode 2 is shown at +1sigma Fig. (b) and -1sigma Fig. (c) and the scores are plotted against the number of ECG major criteria in Fig. (d). Seven other significantly significant correlations were found (p<0.05), three for mode 1 correlated to index 1 (the number of major MRI criteria, p=0.049), index 5 (existence of an ICD, p=0.044) and to index 11 (existence of fibrosis, p=0.016), mode 2 correlated with index 2 (the number of minor MRI criteria, p=0.044), mode 7 correlated with index 7 (syncope episodes, p=0.036) and mode 10 correlated with index 10 (the presence of fat, p=0.019).

**Figure 1:**
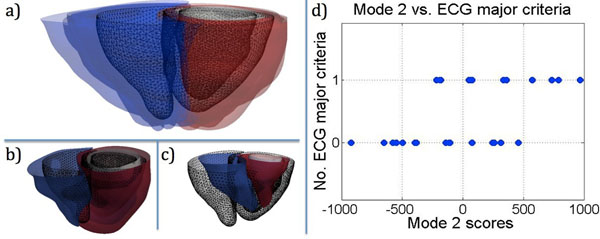
(a) The computed mean (black wireframe) and all subjects (RV endocardium in blue, LV endocardium in white, LV epicardium in white), mode 2 at +1sigma (b) and -1sigma (c), correlation of shape mode 2 and clinical parameter 8: number of ECG major criteria.

## Conclusions

A computational method for analysing shape abnormalities in ARVC patients and correlating these with the 2010TFC indices is presented and applied to 28 patients. The results indicate that the abnormal ventricular structure of ARVC patients may be affected by the clinical symptoms identified by the 2010TFC indices, in accordance with expected relationships observed in clinical practice.

## Funding

This project was carried out as a part of the Centre for Cardiological Innovation, Norway, funded by the Research Council of Norway.

